# Comparison of meat quality, fatty acid composition and aroma volatiles of dry-aged beef from Hanwoo cows slaughtered at 60 or 80 months old

**DOI:** 10.5713/ajas.19.0205

**Published:** 2019-07-01

**Authors:** Dicky Tri Utama, Yeong Jong Kim, Hae Seong Jeong, Juntae Kim, Farouq Heidar Barido, Sung Ki Lee

**Affiliations:** 1 Department of Applied Animal Science, College of Animal Life Sciences, Kangwon National University, Chuncheon 24341, Korea; 2 Faculty of Animal Science, Universitas Brawijaya, Malang 65145, Indonesia; 3 Nutritional Resource Research Institute, Seoul 06747, Korea

**Keywords:** Aroma, Consumer Acceptance, Low-grade Beef, Physicochemical Properties, Tenderness

## Abstract

**Objective:**

The objective of this study was to compare the quality of dry-aged beef from cull Hanwoo cows slaughtered at 60 or 80 months old.

**Methods:**

A total of eight cull Hanwoo carcasses with a quality grade of 3 (low-grade) were selected and divided into two age groups: 63.5±2.5 months old (n = 4) and 87.8±4.5 months old (n = 4). Whole *longissimus thoracis et lumborum* from the 11th rib to the last *lumbar vertebrae*, including the back fat, was removed from the carcass at 24 h postmortem and aged for 50 days in darkness at a temperature of 2°C±1°C, a relative humidity of 85% and an air flow of 2 m/s. The sampling was performed aseptically after 0, 20, 24, 40, and 50 days of aging.

**Results:**

Regardless of the aging period, aging increased the lightness (p<0.05), redness (p<0.05) and yellowness (p<0.05) at initial blooming (90 min after slicing) and the overall acceptance (p<0.05). No further tenderization effect was found after 20 days of aging, but aging for 50 days significantly increased the lipid oxidation (p<0.05). The generation of aroma volatiles in the roast steak from aged samples was higher (p<0.05) than that of non-aged samples. No significant effect of age at slaughter was found on the color, pH, water-holding capacity, cooking loss, shear force value, bacterial counts, volatile basic nitrogen, consumer acceptance, lipid oxidation, fatty acid composition or aroma volatiles.

**Conclusion:**

The quality of dry-aged beef obtained from cull Hanwoo cows slaughtered at either 60 or 80 months old with similar quality grade was comparable and extending dry aging for more than 40 days is not recommended considering the costs and further lipid oxidation.

## INTRODUCTION

In the beef cattle industry, steers are the most slaughtered and are used for producing marbled beef, while cows are mostly used for breeding purposes. The cows are culled when there is a loss of performance to control the production costs [1]. The meat quality of the cull cows is much lower than that of steers, as it has a lower marbling score [2]. Among Korean consumers, highly marbled Hanwoo beef is renowned as the most favorable and exclusive type of beef due to its abundance of intramuscular fat compared with the other breeds available in the Korean market [3]. Marbling in beef has a positive correlation with eating quality attributes such as tenderness, juiciness and flavor [4]. To overcome the weakness of low-marbled beef produced from cull cows, particularly the toughness issue, postharvest methods, such as mechanical tenderization, electrical stimulation and marination, can be applied [5]. However, these attempts have only focused on tenderization. Another attempt to improve the overall palatability, including the flavor, of low-marbled beef and increase a consumer’s willingness-to-pay is through aging [6,7].

There are two general aging methods that have been widely used: wet/vacuum aging and dry aging. For wet aging, primal or subprimal beef cuts are packed into vacuum bags and stored at refrigeration temperatures. For dry aging, primal or subprimal beef cuts are hung or placed on stainless steel gratings in a specialized chamber with controlled temperature, humidity and air flow [8]. The control of humidity and air flow of the aging chamber is not required for wet aging, and thus, the production costs of this aging method are cheaper than those of dry aging and are more widely applied in the meat industry. Among aging methods, dry aging is known to enhance the flavor more than wet aging and increases the willingness-to-pay [6,9]. Bloody and serumy, as well as metallic and sour flavors, are associated with wet-aged beef, while beefy and roasted aromas are associated with dry aging [10]. However, it is not clear whether different aging methods result in different tenderness levels [6,7,9].

In regards to age at slaughter, the quality of dry-aged beef from Hanwoo cattle of different sexes that are slaughtered at different ages, more specifically at 28-month-old for steers and at 48-month-old for cull cows, is not different at all, regardless of the aging method used [11]. To confirm previous results and to provide new information, this study aimed to compare the quality of dry-aged beef from cull Hanwoo cows slaughtered at 63.5±2.5 months old and 87.8±4.5 months old.

## MATERIALS AND METHODS

### Sample preparation

Cull Hanwoo cows were slaughtered at a commercial slaughter plant according to standard operating procedures. The animals were stunned using a captive-bolt stunner prior to slaughter, and the carcasses were not electrically stimulated. The quality and yield grade of the carcasses were evaluated by the Korea Institute for Animal Products Quality Evaluation. A total of eight carcasses with a quality grade of 3 and a yield grade of B were selected and divided into two age groups: 60 mo. (63.5± 2.5 months old, n = 4) and 80 mo. (87.8±4.5 months old, n = 4). Whole *longissimus thoracis et lumborum* from the 11th rib to the last *lumbar vertebrae*, including the back fat, was removed from the carcass at 24 h postmortem and immediately transferred to a commercial dry aging plant. The samples were placed on stainless steel gratings and aged in darkness at a temperature of 2°C±1°C, a relative humidity of 85% and an air flow of 2 m/s. The total aging period lasted 50 days, and sampling was performed on day 0, 20, 24, 40, and 50. For analysis, 12 cm-thick prime cuts were aseptically removed and vacuum-packed after bacterial sampling was performed on the surface of the meat, and they were transferred to the laboratory in an icebox within one hour for analysis. After being removed from the pack, subcutaneous fat and visible connective tissues were trimmed off. A 2 cm-thick steak was sliced for color, pH, water-holding capacity (WHC), lipid oxidation and volatile basic nitrogen analysis. The steak was bloomed for 90 min prior to color measurement. The steak was then ground for analysis. The mean value (and standard error in parentheses) of the moisture content of the prime cuts of each age group on day 0 were 67.1% (0.95) for 60 mo and 67.3% (0.23) for 80 mo, while the fat content was 11.54% (0.90) for 60 mo and 10.27% (0.54) for 80 mo. Another 2 cm-thick steak, which was prepared in duplicate, was sliced for cooking loss and shear force analysis. The remainder of the cuts were stored at −24°C under vacuum for aroma volatiles and sensory analysis. The remainder of the ground samples were lyophilized using a bench-top freeze dryer (Eyela FDU-1200, Tokyo Rikakikai Co., Ltd., Tokyo, Japan). The lyophilized samples were stored at −70°C for fatty acid analysis.

### Instrumental surface color analysis

The color of the bloomed-meat was recorded at five different locations on the surface of the meat by measuring the Commission International De L’eclairage lightness (L*), redness (a*) and yellowness (b*) using a chromameter (CR-400, Konica Minolta Sensing Inc., Tokyo, Japan) with a closed cone attached. The light source of illuminant C (2° observer) with 8 mm aperture was calibrated using a white plate (Y = 93.6, X = 0.3134, y = 0.3194).

### pH and water-holding capacity analysis

The pH was measured in triplicate, sample (5 g) was combined with 45 mL of distilled water then homogenized at 10,000 rpm for 60 s using a homogenizer (PH91, SMT Co., Ltd., Chiba, Japan). The pH value of the meat slurry was recorded using a pH meter calibrated with acid (pH 4.01) and neutral (pH 7.00) technical buffer solutions with an automatic temperature compensation program (SevenEasy pH, Mettler-Toledo GmbH, Greifensee, Switzerland).

The WHC was determined according to the method from Kristensen and Purslow [12] with modifications. The WHC was defined as the proportion (%) of remaining moisture per total original moisture content of fresh ground sample. Briefly, 5 g of ground sample was weighed into graduated centrifuge tubes and prepared in triplicate. The tube was sealed and heated for 30 min in a 75°C water bath. The tubes were cooled in a chilling room (2°C±2°C) for 30 min then centrifuged at 980 *g* at 4°C for 10 min. The supernatant was decanted and measured, and the moisture contents of both raw sample and supernatant were determined according to the AOAC method [13].

### Cooking loss and shear force analysis

The 2.0-cm-thick samples were placed in polyethylene zipper bags, prepared in duplicate and cooked in a water bath at 80°C until the core temperature of 72°C was reached. The cooked samples were then immediately removed from the bags, let rest at room temperature until evaporation was complete, blotted using a kitchen towel and weighed. Cooking loss was expressed as the percentage of weight loss against fresh weight.

After weighing, the cooked samples were cut into cubes (2 cm×2 cm×1 cm; lenth×width× thickness) and subjected to shear force measurement using a TA-XT2*i* Plus (Stable Micro Systems, Godalming, UK). Samples were cut through the slit of the table against the grain as the blade moved down with a constant speed of 200 mm/min [14]. Each assay was repeated eight times for each sample.

### Bacterial counts

Standard method [15] was used for bacterial growth measurement, 1 g of sample taken aseptically from the surface of the meat was put in a sterile bag and prepared in triplicate (Nasco Whirl-Pak, Fort Atkinson, WI, USA). Sample was homogenized with 9 mL sterilized 0.1% (w/v) peptone saline for 2 min using a stomacher (400, Seward Laboratory, Worthing, UK). Decimal dilutions were prepared using sterilized 0.1% (w/v) peptone saline. Total aerobic plate count, lactic acid bacteria and total coliform were enumerated using plate count agar, de Man-Rogosa-Sharpe agar and violet red bile agar, respectively (Difco Laboratories Inc., Livonia, MI, USA). The plates for the enumeration of total aerobic plate count and total coliform were incubated at 37°C for 24 to 48 h, while those for the enumeration of lactic acid bacteria were incubated at 35°C for 48 h. Microbial population was expressed as log colony-forming unit (CFU)/g.

### Volatile basic nitrogen

Sample (5 g) was homogenized (UltraTurrax T25 basic, IkaWerke GmbH and Co., Staufen, Germany) for 1 min with 90 mL of distilled water. The supernatant solution was filtered using a filter paper #1 (Whatman, Maidstone, UK). A 0.01 N of boric acid was placed in the inner section of a Conway micro-diffusion cell (Sibata Ltd., Saitama, Japan). One mL sample solution and 1 mL of saturated K_2_CO_3_ were also placed into the outer section of the same cell, and the lid was immediately closed. The cell was incubated at 37°C for 100 min, and it was then titrated against 0.02 N H_2_SO_4_. The volatile basic nitrogen content was calculated and reported as mg % according to Miwa and Iida [16].

### Sensory evaluation

For sensory evaluation, 25 consumers were participated to evaluate tenderness, juiciness, flavor and overall acceptance of skillet-roasted samples using a seven-point hedonic scale, ranging from very unacceptable (score 1) to very acceptable (score 7) [17]. The frozen steak was thawed overnight in a chilling room (2°C±2°C), sliced into 2 cm thick and roasted until the core temperature of 72°C was achieved using a skillet on a hot plate set at 165°C (Zhongsan Tonsun Electric Appliance Co., Ltd., Guangdong, China). Samples were then rest for 5 min on aluminum foil and cut into 2×1 cm (length× width) prior to serving. Drinking water was provided to cleanse the palate.

### Lipid oxidation

Lipid oxidation was measured using thiobarbituric acid reactive substances (TBARS) method with modifications [18]. Sample (0.5 g) was prepared in triplitcate in 25-mL test tube, vortex-mixed with three drops of antioxidant mixture (consisting of 54% propylene glycol, 40% Tween 20, 3% butylated hydroxytoluene, and 3% butylated hydroxyanisole) and 3 mL of 1% thiobarbituric acid in 0.3% NaOH. Subsquently, 17 mL of 2.5% trichloroacetic acid in 36 mM HCl was added and the tube was closed with cap. The sample was heated in a water bath (BW-20G, Biotechnical Services, Inc., North Little Rock, AR, USA) at 100°C for 30 min and immersed in icy water for 15 min. Aqueous layer (5 mL) was taken and mixed with 3 mL of chloroform and the mixture was centrifuged at 2,400× g for 30 min at 4°C (1248R, Labogene, Lynge, Denmark) to precipitate the dirt (mostly fat) into chloroform layer. The absorbance value of the clear pinkish upper layer was recorded at 532 nm (UV-mini 1240 PC, Shimadzu Corp., Kyoto, Japan) against blank (deionized water was used to replace sample). Data was expressed in mg of malondialdehyde per kg sample.

### Fatty acid composition analysis

The meat fat was extracted from the samples with a chloroform-methanol (2:1 v/v) solution and prepared in triplicate [19]. Fatty acid methyl esters were prepared by mixing the saponified fat with boron trifluoride and then dissolved in hexane. An Agilent gas chromatography system (6890N, Agilent Technologies, Santa Clara, CA, USA) was used for determining fatty acid composition. The sample in hexane (1 μL) was injected into the GC port by the auto sampler (7683, Agilent Technologies, USA). The inlet temperature was set at 250°C with a split ratio of 100:1. Fatty acid methyl esters were separated using a WCOT-fused silica capillary column (100 m× 0.25 mm i.d., 0.20 μm film thickness; Varian Inc., Palo Alto, CA, USA) with a 1.0 mL/min helium flow. The oven was programmed as follows: 150°C/1 min, 150°C to 200°C at 7°C/min, 200°C/5 min, 200°C to 250°C at 5°C/min, and 250°C/10 min. The temperature of the detector was set at 280°C. The fatty acid peaks were identified using the retention time of fatty acid standards (47015-U, Sigma-Aldrich Corp., LLC., St. Lois, MO, USA). The peak area of each identified fatty acid was used to calculate the proportion (%) against the total identified peak area.

### Aroma volatiles analysis

About 20 g of roasted sample taken from sensory analysis was ground and 3 g of ground sample prepared in duplicate was immediately put in 50-mL headspace vial for volatiles extraction. The volatile compounds from cooked samples were separated and identified by gas chromatography-mass spectrometry (GC-MS) using a modified version of the method described in Ba et al [20] and was used in previous work [21]. Prior to extraction, the temperature of the sample was calibrated to 60°C in a drying oven for 10 min, and carboxen/polydimethylsiloxane fiber (Supelco, Sigma-Aldrich Corp., LLC., USA) with a 75 μm diameter was injected into the vial for extraction for another 30 min. Following extraction, the fiber was injected into the GC port (inlet), which was set to 250°C, and the volatile compounds were desorbed for 5 min at a 1:5 split ratio. Separation was performed using a DB5 fused silica column (30 m×0.25 mm inner diameter, 0.25 μm film thickness, J&W Scientific, Folsom, CA, USA) in a gas chromatograph (7890A Agilent Technologies, USA). The GC oven was programmed to operate at an initial temperature of 40°C for 2 min, increasing to 160°C (at 5°C/min), then to 180°C (at 6°C/min, holding time of 5 min), and finally to 200°C (at 10°C/min, holding time of 5 min). The interface and quadruple temperatures were 280°C and 150°C, respectively. Helium was used as the carrier gas with a flow rate of 1 mL/min. Volatile compounds were detected using a mass spectrometer (5975C, Agilent Technologies, USA). The ion source temperature of the MS was set to 280°C with an electron impact of 70 eV. A scanning mass range of 50 to 450 m/z with a scan rate of 1 scan/s was used. Tentative identification was performed by comparing the experimental spectra to the National Institute of Standards and Technology Mass Spectral Library. Data are presented as peak area unit (AU) ×10^6^/g.

### Statistical analysis

Two way analysis of variance was used for determining the effect of age at slaughter, aging period and their interactions. When significant effect was observed, the mean values were then separated by Duncan’s multiple range test at the 5% level of significance. Data for total coliform from day 0 (not detected) were adjusted to 0.01 log CFU/g prior to analysis. Analyses were performed using R-version 3.3.3 [22] with “agricolae” package for Duncan’s multiple range test [23].

## RESULTS

### Meat color

Aging increased the instrumental surface color, including lightness (p<0.05), redness (p<0.05) and yellowness (p<0.05), at initial blooming (90 min after slicing) compared to non-aged samples (Table 1). There were no significant differences in color among all aged samples obtained from different aging periods. Regarding the age at slaughter, no significant differences were found.

### pH, water-holding capacity, cooking loss, and shear force value

The effect of the age at slaughter and aging period on meat quality is shown in Table 2. No significant effect of the age at slaughter was found on pH, WHC, cooking loss, and shear force value. The meat quality of dry-aged beef obtained from cull Hanwoo cows slaughtered at either 60 or 80 months old was comparable. However, the effect of the aging period on those variables was obvious. Dry aging for 50 days significantly increased the pH (p<0.05). However, no significant changes in pH were found from the beginning of aging to day 40. The highest WHC of the meat was found at the beginning of aging and the capacity decreased after 20 days of aging (p<0.05). The lowest WHC was observed on day 50 of aging (p<0.05). There were no significant differences in cooking loss from the beginning of aging to day 24, but it decreased after day 40. Aging significantly (p<0.05) reduced the shear force value or tenderized the meat. However, no significant differences were found for the shear force value among aged samples taken from different aging periods.

### Bacterial counts and freshness

There was no significant effect of the age at slaughter on any bacterial counts (Table 3). Prolonging the aging period increased the number of total aerobic plate count (p<0.05) and total coliforms (p<0.05), but the number of lactic acid bacteria was not affected. The freshness of the meat was maintained across 50 days of dry aging, although this dry-aging period increased the generation of volatile basic nitrogen (p<0.05). No significant effect of the age at slaughter was found on the amount of volatile basic nitrogen (Table 3).

### Consumer acceptance

Dry aging, regardless of the aging period, increased the score of tenderness (p<0.05) and overall acceptance (p<0.05). Aging did not affect the score of juiciness and flavor (Table 4). This indicates that panelists did not notice the aroma differences between samples obtained from different aging periods. No significant effect of age at slaughter was found for any of the variables. Therefore, the dry-aged beef obtained from either 60-month-old or 80-months-old Hanwoo cows are comparable and still acceptable.

### Lipid oxidation, fatty acid composition, and aroma volatiles

**Figure 1:**
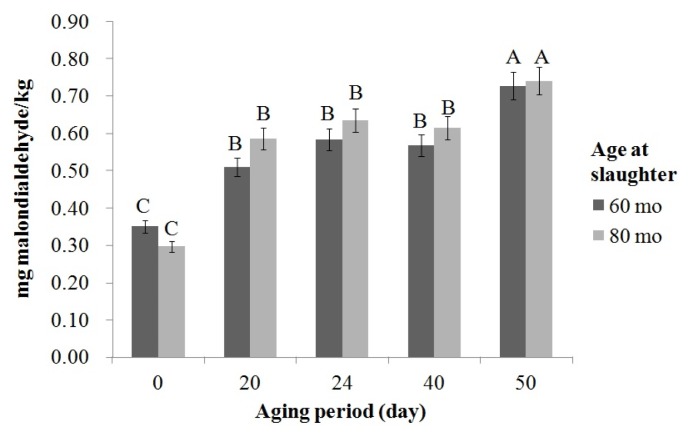
Changes in thiobarbituric acid reactive substances value (mg malondialdehyde/kg) in beef from cull cow slaughtered at different ages during dry aging. ^A–C^ Mean values are significantly different according to aging period (p<0.05).

There was no significant effect of age at slaughter on the occurrence of lipid oxidation during aging. The longer the aging period, the higher the amount of malondialdehyde or the lipid oxidation product observed (Figure 1). The highest TBARS value was observed on day 50 of aging (p<0.05), while there were no significant differences in the TBARS value between aged samples taken from day 20, 24, and 40.

The changes in fatty acid composition were observed after 40 days of aging (Table 5), particularly the reducing proportion (p<0.05) of oleic acid (C18:1n9), gamma-linolenic acid (C18:3n6), and alpha-linolenic acid (C18:3n3), and the increasing proportion (p<0.05) of arachidonic acid (C20:4n6). The declining proportion of monounsaturated fatty acid (MUFA) and n3 polyunsaturated fatty acid (PUFA) resulted in an increasing proportion of n-6 PUFA and thus increased the n6 to n3 PUFA ratio (p<0.05).

Although the sensory evaluation did not reveal that dry-aged beef significantly improved the acceptance of flavor, instrumental results obtained from gas chromatography revealed the opposite. Dry aging, particularly after 40 days, enhanced the release of all identified volatile compounds from the roast sample (p<0.05). No significant effect of age at slaughter was found on the generation of all identified aroma volatile compounds (Table 6).

## DISCUSSION

Aging was found to increase the color of meat at initial blooming in this study. Meat color is affected by two mechanisms of mitochondrial activity; oxygen consumption and metmyoglobin reducing activity [24]. Aging increases the initial red color intensity as the mitochondrial ability to consume oxygen is reduced by postmortem aging [25,26]. Higher oxygen consumption by the mitochondria in non-aged beef steak inhibits myoglobin oxygenation and thus decreases the formation of oxymyoglobin or maintains myoglobin in the deoxy state; in other words, it decreases the intensity of the red color [27]. Aging seems to provide benefits to enhance the red color intensity of the meat at initial blooming; however, previous studies have revealed that the instrumental and visual bright red color of aged-beef steak during display under highly oxygenated (80%) modified atmosphere packaging (MAP) decreased faster than non-aged steak after six days as the mitochondrial-related metmyoglobin reducing activity declines during aging [27,28]. Therefore, aged beef steak should be sold before six days of display under highly oxygenated MAP.

The structural breakdown of meat protein complexes during aging is due to the activity of endogenous proteases, such as calpains/calpastatins and cathepsins/cystatins, and this phenomenon causes not only meat tenderization but also the declining ability of meat protein to bond moisture [29]. Therefore, the dry-aged samples in this study lose their WHC, which results in higher cooking loss than non-aged samples. No further tenderization was observed after 20 days of aging, as the activity of the lysosomal and cytosolic cathepsins, including cathepsin B and cathepsin B+L, reached the optimum level within the first 14 days postmortem and decreased afterwards [30].

According to the observed number of bacterial counts (aerobic bacteria of less than 7 log CFU/g) and Processing Standard and Ingredient Specifications for Livestock Products [31], the dry aging of the whole *longissimus thoracis et lumborum* cut from cull Hanwoo cows slaughtered at either 60 or 80 months and for up to 50 days is still appropriate. Although dry aging for 50 days increased the generation of volatile basic nitrogen, the number was still below the limit of 20 mg % according to the Korean Processing Standard and Ingredient Specifications for Livestock Products [31].

In this study, lipid oxidation affected the shift in fatty acid composition and particularly C18:1n9, C18:3n6, C18:3n3, and C20:4n6. The declining proportion of oleic acid after dry aging was also observed in a previous study [32]. The extension of the aging period could be responsible for the oxidation of C18:3n6 and C18:3n3 and the declining proportion of MUFA and n3 PUFA, which results in the increase of the n6 to n3 ratio and particularly the increase of the C20:4n6 proportion. An n3 fatty acid contains carbon double bonds, which weaken the structure and make it prone to oxidation [33].

Regardless of the age of the animal, these results also confirm that dry aging intensifies the flavor of beef. Untrained panelists could not determine aroma differences between obtained samples before and after aging in this study. However, highly trained panelists in previous study confirmed that dry aging intensifies several flavor attributes [10]. Aging enzymatically breaks down meat proteins into smaller structures (oligopeptides, peptides and free amino acids) and oxidizes the meat fat, thus providing precursors for the generation of thermally induced aroma volatile compounds [8]. Lee et al [34] found that dry aging increases the amount of total free amino acids including those associated with bitterness and sweetness. These free amino acids also contribute to the generation of aroma volatile compounds as precursors. Hexanal was the most abundant volatile compound and was followed by decane, octanal, and hexane. These volatile compounds are mainly the product of lipid oxidation and contribute to the sensation of the fatty and oxidized aroma [35]. Sulfur-containing compounds and furans that are associated with beef aroma were also identified as having higher levels in dry-aged samples than non-aged ones. Benzothiazole and thiazole are strongly associated with meat aroma and the products of the thermal degradation of sulfur-containing amino acids, e.g., cystine and methionine [33,35]. Benzaldehyde was also found to be higher in dry-aged samples compared non-aged samples. The Maillard reaction that occurs between reducing sugars and sulfur-containing amino acids results in the generation of the key odorants of meat flavor, while α-amino acids, e.g., leucine, phenylalanine, cysteine and methionine, can also degrade and react with lipid oxidation-related aldehydes through Strecker’s degradation to form volatile compounds with sweet aroma characteristics, e.g., benzaldehyde [33,35].

## CONCLUSION

Dry aging is approved to enhance the quality of low-grade beef from cull Hanwoo cows in this study. Extending dry aging for more than 40 days does not seem necessary to further improve the quality and acceptance of beef from cull Hanwoo cows slaughtered at either 60 or 80 months old. Considering the production cost and lipid oxidation, dry aging for up to 40 days is sufficient to achieve an acceptable quality for aged-beef from cull cows regardless of the age at slaughter. The quality was the same for dry-aged beef obtained from cull Hanwoo cows slaughtered at either 60 or 80 months old with similar quality grades. Therefore, dry aging can be applied to both age groups with comparable results.

## Figures and Tables

**Table 1:** Changes in surface color of beef from culled Hanwoo cows slaughtered at different ages during dry aging

Variable	Age (month)	Aging period (d)					SEM

		0	20	24	40	50	
Lightness (L*)	60	36.0^B^	39.0^A^	41.0^A^	41.0^A^	40.3^A^	0.61
	80	35.0^B^	38.7^A^	38.4^A^	39.7^A^	39.2^A^	0.53
Redness (a*)	60	21.7^B^	22.3^A^	24.8^A^	25.2^A^	25.9^A^	0.52
	80	21.9^B^	22.9^A^	24.5^A^	24.4^A^	24.1^A^	0.33
Yellowness (b*)	60	11.6^B^	11.8^A^	13.5^A^	13.7^A^	14.7^A^	0.38
	80	11.8^B^	12.6^A^	12.3^A^	13.0^A^	13.6^A^	0.20

Data are presented as mean value (standard error).

SEM, standard error of the mean.

A–C Mean values are significantly different according to aging period (p<0.05).

**Table 2:** Changes in meat quality of beef from culled Hanwoo cows slaughtered at different ages during dry aging

Variable	Age (month)	Aging period (d)					SEM

		0	20	24	40	50	
pH	60	5.60^B^	5.60^B^	5.61^B^	5.63^B^	5.69^A^	0.01
	80	5.57^B^	5.57^B^	5.54^B^	5.56^B^	5.69^A^	0.02
Water-holding capacity (%)	60	64.2^A^	50.2^B^	51.0^B^	50.6^B^	46.1^C^	1.78
	80	65.5^A^	51.7^B^	50.8^B^	49.7^B^	47.1^C^	1.86
Cooking loss (%)	60	27.6^B^	30.9^B^	30.7^B^	33.1^A^	33.5^A^	0.61
	80	29.2^B^	30.5^B^	30.8^B^	33.6^A^	33.4^A^	0.55
Shear force (kg)	60	6.16^A^	2.59^B^	2.00^B^	1.80^B^	1.84^B^	0.48
	80	6.93^A^	3.35^B^	2.24^B^	2.19^B^	2.06^B^	0.53

Data are presented as mean value (standard error).

SEM, standard error of the mean.

A–C Mean values are significantly different according to aging period (p<0.05).

**Table 3:** Changes in bacterial counts on beef from culled Hanwoo cows slaughtered at different ages during dry aging

Variable	Age (month)	Aging period (d)					SEM

		0	20	24	40	50	
Total plate count (log CFU/g)	60	1.88^C^	3.78^B^	4.66^A^	4.60^A^	5.38^A^	0.35
	80	1.89^C^	3.86^B^	4.01^AB^	4.17^AB^	4.36^A^	0.26
Lactic acid bacteria (log CFU/g)	60	1.65	2.26	1.68	1.69	1.41	0.08
	80	1.73	2.29	1.70	1.76	1.21	0.10
Total coliform (log CFU/g)	60	ND^C^	1.58^B^	2.81^A^	2.97^A^	3.30^A^	0.19
	80	ND^C^	1.60^B^	2.56^A^	2.76^A^	3.24^A^	0.18
Volatile basic nitrogen (mg %)	60	9.13^B^	11.02^A^	11.65^A^	13.46^A^	13.22^A^	0.51
	80	8.50^B^	10.08^A^	12.99^A^	12.75^A^	13.70^A^	0.64

Data are presented as mean value (standard error).

SEM, standard error of the mean; CFU, colony-forming unit; ND, not detected (data were adjusted to 0.01 log CFU/g prior to statistical analysis).

A–C Mean values are significantly different according to aging period (p<0.05).

**Table 4:** Consumer acceptance score of roasted beef from culled Hanwoo cows slaughtered at different ages and dry-aged for different periods

Variable	Age (month)	Aging period (d)					SEM

		0	20	24	40	50	
Tenderness	60	3.30^B^	5.20^A^	4.70^A^	5.10^A^	4.60^A^	0.20
	80	3.20^B^	5.20^A^	5.40^A^	5.10^A^	5.10^A^	0.23
Juiciness	60	4.10	4.90	4.40	4.70	4.60	0.08
	80	4.00	5.00	5.50	4.70	5.20	0.15
Flavor	60	4.70	4.30	4.90	4.70	5.20	0.08
	80	4.60	4.70	5.30	4.60	5.10	0.08
Overall	60	4.10^B^	4.60^AB^	4.80^A^	4.80^A^	4.90^A^	0.08
	80	3.70^B^	5.10A	5.60^A^	4.80^A^	5.10^A^	0.18

Data are presented as mean value (standard error).

SEM, standard error of the mean.

A,B Mean values are significantly different according to aging period (p<0.05).

**Table 5:** Changes in fatty acid composition of beef from culled Hanwoo cows slaughtered at different ages before and after adry aging

Fatty acid	60 months old		80 months old		SEM
	
	Day 0	Day 40	Day 0	Day 40	
C14:0	3.21	3.23	2.95	3.53	0.04
C16:0	28.2	29.9	28.1	30.9	0.17
C16:1n7	5.16	4.91	4.49	5.06	0.10
C18:0	12.0	12.8	12.5	13.4	0.08
C18:1n9	49.2^A^	46.3^B^	49.8^A^	44.6^B^	0.12
C18:2n6	1.66	2.13	1.51	1.99	0.04
C18:3n6	0.11^A^	0.07^B^	0.10^A^	0.06^B^	0.01
C18:3n3	0.37^A^	0.26^B^	0.37^A^	0.27^B^	0.02
C20:4n6	0.09^B^	0.23^A^	0.12^B^	0.19^A^	0.00
C20:5n3	0.04	0.08	0.05	0.06	0.00
SFA	43.5	46.0	43.6	47.8	0.14
MUFA	54.6^A^	51.2^B^	54.2^A^	49.6^B^	0.14
PUFA	2.06^B^	2.71^A^	2.06^B^	2.51^A^	0.04
n6	1.65	2.37	1.63	2.18	0.04
n3	0.41^A^	0.34^B^	0.42^A^	0.33^B^	0.02
n6/n3	3.54^B^	7.37^A^	3.88^B^	6.83^A^	0.29

Data are presented as mean value (standard error).

SEM, standard error of the mean; SFA, saturated fatty acids; MUFA, monounsaturated fatty acids; PUFA, polyunsaturated fatty acids.

A,B Mean values are significantly different according to aging period (p<0.05).

**Table 6:** Aroma volatiles (area unit ×10^6^/g) released from the roasted beef from culled Hanwoo cows slaughtered at different ages before and after adry aging

Compound name	60 months old		80 months old		SEM
	
	Day 0	Day 40	Day 0	Day 40	
2-Butene	1.57^B^	3.21^A^	1.55^B^	3.02^A^	0.25
Hexane	2.50^B^	4.25^A^	2.45^B^	4.40^A^	0.29
2-Methylfuran	1.58^B^	2.57^A^	1.66^B^	2.67^A^	0.17
Thiazole	1.59^B^	2.48^A^	1.56^B^	2.62^A^	0.16
3-Methylbutanal	0.24^B^	1.39^A^	0.24^B^	1.43^A^	0.17
2-Methylbutanal	0.55^B^	2.57^A^	0.57^B^	2.45^A^	0.29
Hexanoic acid	0.25^B^	0.49^A^	0.24^B^	0.47^A^	0.04
Toluene	0.54^B^	0.84^A^	0.51^B^	0.84^A^	0.05
Octane	0.57^B^	1.36^A^	0.58^B^	1.37^A^	0.12
Hexanal	16.0^B^	34.5^A^	14.4^B^	32.5^A^	2.82
Heptanal	2.77^B^	4.50^A^	2.74^B^	4.59^A^	0.28
Benzaldehyde	0.82^B^	1.22^A^	0.85^B^	1.29^A^	0.06
Benzothiazole	0.43^B^	0.83^A^	0.42^B^	0.90^A^	0.07
Decane	3.40^B^	7.43^A^	3.32^B^	7.49^A^	0.61
Octanal	3.38^B^	5.26^A^	3.39^B^	5.59^A^	0.31
Nonanal	0.35^B^	0.57^A^	0.35^B^	0.54^A^	0.03

Data are presented as mean value (standard error).

SEM, standard error of the mean.

A,B Mean values are significantly different according to aging period (p<0.05).
